# Network pharmacology of cellular targets in major depressive disorder and differential mechanisms of fluoxetine, ketamine and esketamine

**DOI:** 10.1016/j.csbj.2025.12.023

**Published:** 2025-12-29

**Authors:** Silvia Tapia-Gonzalez, Josué García Yagüe, George E. Barreto

**Affiliations:** a Grupo de Neurofisiología Celular, Departamento de Ciencias Médicas Básicas, Facultad de Medicina, Instituto de Medicina Molecular Aplicada-Nemesio Díez (IMMA-ND), Universidad San Pablo-CEU, CEU Universities, Urbanización Montepríncipe s/n, Madrid, Spain; b Department of Biological Sciences, University of Limerick, Limerick, Ireland

**Keywords:** Major depressive disorder, NFKB, OPRM1, GSK3B, Fluoxetine, Ketamine, Esketamine, Network pharmacology

## Abstract

Major depressive disorder (MDD) is a multifactorial mental health condition involving genetic, environmental, and neurobiological factors. Conventional antidepressants such as fluoxetine, a selective serotonin reuptake inhibitor, require weeks to exert therapeutic effects, whereas ketamine and esketamine act rapidly via glutamatergic modulation. These drugs may also converge on the inhibition of glycogen synthase kinase 3 beta (*GSK3B*) as a key mechanism for their antidepressant effects, increasing neuroplasticity, synaptic transmission, and neuronal survival through upregulation of brain-derived neurotrophic factor (*BDNF)*. Part of the antidepressant effects of ketamine also seems to depend on opioid receptor activation. Despite recent progress, variability in antidepressant response in MDD remains unclear. This work explores, via meta-analysis and network fragility analysis, key molecular mechanisms in MDD, how these drugs exert actions, and highlights potential therapeutic targets for MDD. We performed a network pharmacology approach to unravel the key cellular processes involved in MDD, including altered synaptic plasticity, neurogenesis, apoptosis, and neuroinflammation. Second, we explored the therapeutic role of these treatments on these altered cellular processes. By integrating drug-target data with MDD-associated genes, we identified the opioid receptor mu 1 (*OPRM1*), epidermal growth factor receptor (*EGFR*) and *GSK3B* as key druggable targets. Network analysis further suggested that nuclear factor kappa B (*NFKB*) may regulate all three, positioning it as a central node linking inflammation, synaptic plasticity, and neuronal metabolism in MDD. We hypothesize that targeted modulation of these genes may optimize the therapeutic efficacy, while *NFKB* emerges as a promising candidate biomarker for guiding treatment strategies in MDD.

## Graphical Abstract

**Figure:**
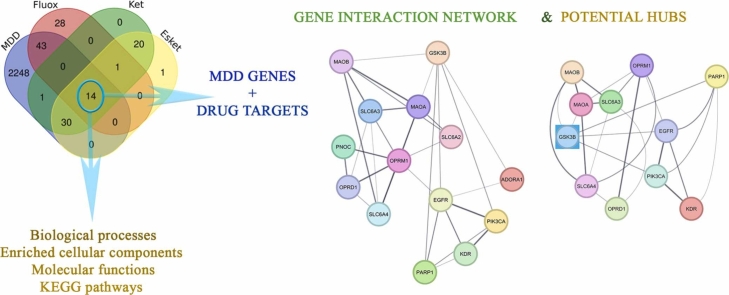

## 1 Introduction

Major depressive disorder (MDD) is a debilitating mental health condition characterized by persistent sadness, anhedonia, and cognitive impairment. MDD affects millions globally, with a significant portion of patients exhibiting resistance to standard treatments [1]. Its multifactorial etiology involves genetic, environmental, and neurobiological factors [1]. The pathophysiology of MDD involves a dysfunction in multiple cellular processes, including disruption of neurogenesis [2] and synaptic plasticity [3], [4] as well as an increase in neuroinflammatory signalling and apoptosis [5], [6].

In the central nervous system (CNS), many of these functions are controlled by *NFKB*, which plays a relevant role in synaptic plasticity and memory and is associated with the expression of inflammatory genes such as interleukin-1β (IL-1β), interleukin-6 (IL-6), tumour necrosis factor-α (TNF-α), cyclooxygenase-2 (COX-2) and inducible nitric oxide synthase (iNOS) in astrocytes and microglia [7], [8]. Its regulation is mediated by mitogen-activated protein kinases (MAPKs) such as p38 MAPK (MAPK14) and c-Jun N-terminal kinases (JNK), whose phosphorylation occurs in situations of stress and inflammation [9]. The inhibition of NFKB in transgenic mice induces a decrease in the gene expression of the enzyme glutamate decarboxylase 65 (GAD65), which catalyzes the production of γ-aminobutyric acid (GABA) in GABAergic interneurons, and glutamate receptor 1 (GluR1) in glutamatergic neurons, producing neuronal hyperexcitability, hyperactivity, seizures, and an improvement in learning [10]. The reduction of GAD65 has also been associated with greater anxiety in rodents and a lower response to anxiolytics such as benzodiazepine diazepam and barbiturate pentobarbital, which act through γ-aminobutyric acid type A (GABA-A) receptors in the presence of GABA [11]. Furthermore, endogenous opioid receptors (OR) (i.e., delta (DOR), mu (MOR), kappa (KOR), and nociceptin/orphanin FQ (NOP), which are involved in reward processing and mood control, may also be putative targets for treating MDD [12], [13], [14]. Among them, MOR1, encoded by the *OPRM1* gene, has been most closely linked to MDD [15], while antidepressant properties of the DOR receptor have also been recently described through disinhibition of pyramidal neurons in the prefrontal cortex [16]. Additionally, EGF has been associated with cellular proliferation and plasticity [17], however, its dysregulation, may contribute differently at various stages of depression [18].

Traditional antidepressants, including selective serotonin reuptake inhibitors (SSRIs), have been the mainstay of treatment but often require several weeks to achieve clinical efficacy [19], [20]. Fluoxetine, one of the most widely prescribed SSRIs, works by blocking the reuptake of serotonin, thereby increasing its synaptic availability. Its relatively favorable side-effect profile and efficacy in a range of depressive disorders have made it a cornerstone of depression treatment [21]. However, fluoxetine’s mechanism is inherently gradual, and its reliance on monoaminergic modulation may not address the full spectrum of depression’s pathophysiology. This delay in response, coupled with treatment resistance in a subset of patients, underscores the need for novel approaches [1].

A delayed response to conventional MDD treatment prolongs suffering and heightens the risk of suicide. Ketamine, traditionally used as an anesthetic, has garnered significant attention for its rapid antidepressant effects [22]. Administered at subanesthetic doses, ketamine acts primarily as an N-methyl-D-aspartate (NMDA) receptor antagonist [2], [23] and has a preferential action in GABAergic inhibitory interneurons [24], [25]. A reduction in GABAergic neurotransmission leads to a loss of inhibition over excitatory glutamatergic neurons, resulting in bursts of glutamate release that active post-synaptic α-amino-3-hydroxy-5-methyl-4-isoxazoleproprionic acid (AMPA) receptors. This modulation of glutamate signalling appears to trigger a cascade that promotes synaptic plasticity and rapid synaptogenesis [26], [27], [28]. Building on these findings, esketamine - the S-enantiomer of ketamine- has been developed and approved as a nasal spray for treatment-resistant depression, offering a new therapeutic option that can produce rapid symptomatic relief [29].

The rapid action of ketamine and esketamine is thought to extend beyond simple NMDA receptor antagonism. Emerging evidence indicates that these agents may influence several intracellular pathways, including the inhibition of GSK3B [30]. This inhibition is posited to contribute to the rapid modulation of mood by enhancing neuroplasticity and attenuating inflammatory signals [31], [32]. Interestingly, fluoxetine, ketamine, and esketamine converge on the inhibition of GSK3B as a key mechanism for their antidepressant effects [30], [33], [34], [35], [36]. Specifically, fluoxetine modulates GSK3B through the activation of the serotonin type 1A (5-HT1A) receptor, which activates protein kinase B (Akt), which phosphorylates GSK3B at ser9, inhibiting it. This suppression allows the activation of β-catenin and the increase in *BDNF* expression and neurogenesis [33], [35], [37]). On the other hand, ketamine and esketamine rapidly suppress GSK3B through NMDA receptor antagonism and subsequent Akt/mTOR (mecanistic target of rapamycin) signalling, which contributes to *BDNF* upregulation and synaptogenesis[34], [36], [38]). Taken together, fluoxetine, ketamine, and esketamine may exert a common antidepressant action by increasing neuroplasticity, synaptic transmission, and neuronal survival through the upregulation of *BDNF* [38]). However, there are also important divergences between these drugs in terms of the signaling pathways they modulate, for example, it has been postulated that part of the antidepressant actions of ketamine would be through its modulation of opioid receptors [39].

Therefore, the aim of this work is, first, to perform a meta-analysis to unravel the cellular processes and key molecules involved in the pathophysiology of MDD, including molecules implicated in altered synaptic plasticity, neurogenesis, apoptosis, and neuroinflammation. Second, to explore the cellular and genetic mechanisms by which fluoxetine, ketamine, and esketamine exert their therapeutic actions in MDD. Finally, by using network fragility analysis, we identified key hubs for MDD and its therapeutics.

## 2 Materials and methods

### 2.1 List of genes modulated in MDD by pharmacological compounds

We adopted a rigorous multidisciplinary strategy to identify genes associated with major depressive disorder (MDD) and assess their potential pharmacological modulators. Initially, data were extracted from the Comparative Toxicogenomics Database (CTD; accessed on 06/11/2024) using the term “major depressive disorder” to obtain all reported associations with the condition. To minimize the inclusion of false positives or redundant entries caused by synonyms, the dataset was validated and refined through cross-referencing with two complementary repositories, MalaCards and Open Targets (accessed on 11/06/2024, which were consulted simultaneously. Gene identifiers were unified, and duplicates eliminated via UniProt. Only genes present in at least two out of three sources were retained for further analysis.

To identify the molecular targets of fluoxetine, ketamine, and esketamine, their SMILES structures were retrieved from PubChem and analyzed with three predictive tools based on molecular similarity and machine learning: Swiss Target Prediction, Similarity Ensemble Approach (SEA) (accessed on 06/11/2024, and TargetNet (accessed on 06/11/2024). The resulting gene lists were compared with the genes associated with MDD to identify pharmacological targets most relevant to the disorder. Finally, the overlapping genes were used to build interaction networks in Cytoscape software (v.3.10), which were analyzed for topology and fragility to reveal key signaling pathways and central nodes involved in how these drugs may affect MDD pathophysiology.

### 2.2 Functional enrichment

To gain deeper insight into the genetic regulation of biological pathways disrupted in MDD and how these processes are modulated by different drugs, we performed a functional enrichment analysis. The parameters used included biological processes, molecular function, and cellular compartments, using the Database for Annotation, Visualization, and Integrated Discovery (DAVID) tool [40], [41]. This platform enables to identify biologically enriched terms and discover functionally related genes, thereby facilitating the detection of patterns of similarities and differences between different experimental conditions. In addition, by grouping redundant annotation terms, DAVID provides a comprehensive and enriched functional annotation, which aids in elucidating the biological significance of the genes involved. Finally, the dot plots graphs were generated using Graphad prism 10.

### 2.3 Meta-analysis of clustered biological enriched terms

To perform a comprehensive functional analysis of the biological processes and molecular pathways associated with the genes identified in all the lists analysed (MDD, fluoxetine, ketamine, esketamine and common genes), we conducted a meta-analysis of the enriched terms. The aim was to integrate and highlight the most relevant and critical terms that reflect the potential impact of the compounds on MDD-related genes.

To this end, we used the Metascape platform (version 3.5) [42], an online tool that combines multiple recognized databases, such as KEGG (Kyoto Encyclopaedia of Genes and Genomes) and Reactome [43], among others. This platform automates the identification and grouping of enriched terms, minimizing redundancies through an approach similar to that used by DAVID. The analysis is based on a similarity calculation using the Kappa coefficient [44], which allows related terms to be grouped into hierarchical clusters. A similarity threshold of 0.3 was employed in our analysis, ensuring that only terms with similar biological meaning were grouped together. Within each cluster, the term with the most significant p-value was considered representative of the group. As a result, a functional list containing the most relevant gene clusters was generated, allowing us to identify which biological pathways and molecular functions are potentially modulated by the compounds analyzed. This functional mapping provides an integrated view of how different drugs could act on genes associated with MDD, thereby contributing to the identification of potential targets for future mechanistic studies.

### 2.4 Network analysis

With the aim of identifying the genes with the greatest functional and structural relevance within the interaction network, we constructed a protein-protein interaction (PPI) network using Cytoscape [45], [46], [47] from the final list of genes obtained from the analysed databases. The main purpose was to detect key genes (hubs), whose presence guarantees the stability and connectivity of the network, and to identify highly connected subgroups using the Molecular Complex Detection (MCODE) algorithm [48].

First, we used the integrated STRING plugin in Cytoscape, with a confidence cutoff of 0.4 and without adding additional interactors, using *Homo sapiens* as the reference organism. STRING is recognised as one of the most reliable metadatabases for integrating experimental and predictive information on interactions between proteins and genes. In addition, it allows these interactions to be associated with the sublocalisation of elements in tissues and cell types, making it a key tool for evaluating the functional impact of networks in specific cellular contexts.

To complement the PPI network analysis and identify topological parameters that would allow us to detect cellular hubs, we used the network analysis tool incorporated in Cytoscape. This tool provides fundamental metrics, including degree (DG), betweenness centrality (BS) and closeness centrality (CS). Degree measures the number of direct interactions of a node; betweenness quantifies the importance of a node as an intermediary in the flow of information in the network [49]; closeness centrality evaluates the average distance of the node from the others, reflecting the efficiency of information propagation [50].

Finally, the MCODE algorithm was applied to identify relevant subclusters within the network, thus allowing functionally coherent molecular complexes to be extracted. MCODE generates subclusters based on various topological criteria and identifies the seed, i.e., the most central and functionally critical node within each module was identified, along with the genes comprising these modules. These clusters provide a refined perspective on the emerging functional architecture of the interaction network and serve as a foundation for future experimental investigations.

#### 2.4.1 Network fragility analysis

To assess the importance of *OPRM1*, *EGFR*, and *GSK3B* in the stability of the node-node interaction network, we performed a fragility analysis by sequentially removing these critical nodes. We started with the complete network, constructed in Cytoscape from the final set of genes of interest. We calculated basic topological metrics using Cytoscape's integrated tools. This included network diameter, defined as the longest shortest path between any two nodes, and reflects the maximum communication distance, while mean path length represents the average shortest path between all node pairs and indicates overall efficiency of information flow. In addition to these metrics, we also assessed clustering coefficient, which measures the tendency of nodes to form tightly connected groups and represents local cohesion. Network density is the ratio to possible connections and shows how interconnected the network is. Finally, heterogeneity quantifies variability in node connectivity and indicates whether the network is dominated by a few highly connected hubs or is more evenly distributed.

Next, we removed each of the three genes identified as hubs according to degree, betweenness and closeness centrality analyses and generated modified networks for each case. After each removal, we recalculated all topological metrics with the same parameters used in the original network. This allowed us to quantify the specific impact of each exclusion and compare the values obtained before and after removal, evaluating the degree of disturbance in terms of communication efficiency (increase in diameter and average path length) and local cohesion (reduction in clustering coefficient and density).

### 2.5 Analysis of the transcriptional regulation of *OPRM1*, *EGFR* and *GSK3B*

**Fig. 1:**
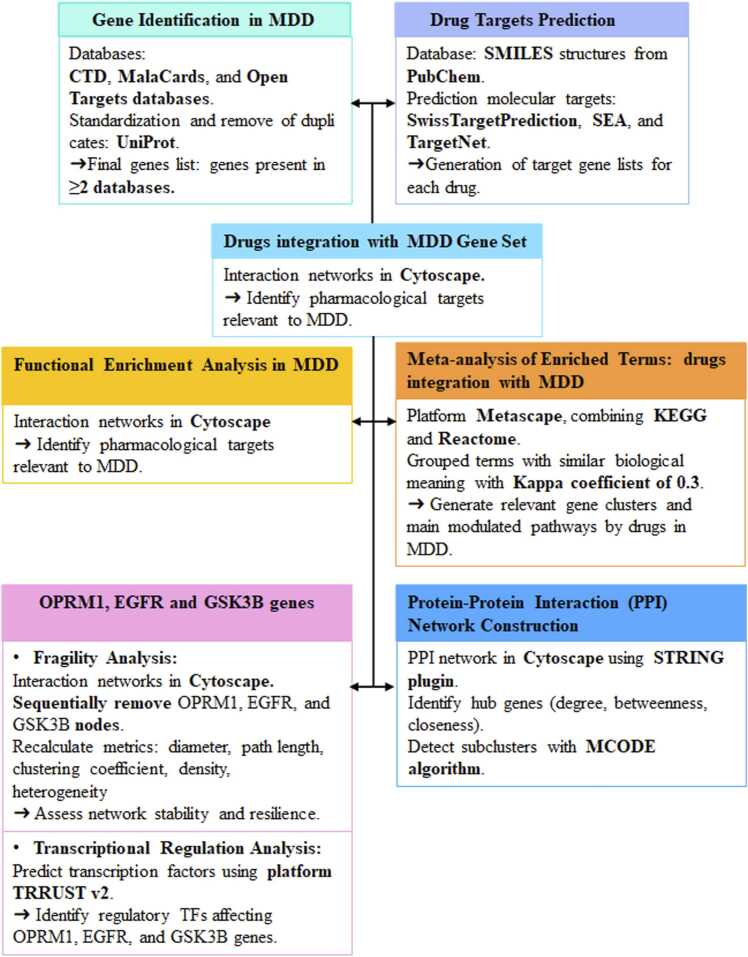
Workflow summarizing the steps used to identify and characterize MDD-related genes, predict drug targets, perform enrichment and meta-analyses, and construct and analyze the protein–protein interaction (PPI) network, including fragility and transcriptional regulation analyses. Abbrs: CTD: Comparative Toxicogenomics Database; MalaC: MalaCards; OpenT: Open Targets.

To identify transcription factors (TFs) potentially involved in the regulation of the genes of interest -*OPRM1*, *EGFR*, and *GSK3B*- we performed a transcriptional regulation prediction analysis using the platform TRRUST v2 (Transcriptional Regulatory Relationships Unraveled by Sentence-based Text mining) [51]. TRRUST is a manually curated database that compiles TF-gene interactions extracted from scientific literature based on experimental evidence, making it a robust tool for studying regulatory networks. The list of differentially expressed genes in the analysed datasets was used as input, prioritizing those with functional relevance in neuroplasticity, synaptic metabolism, and inflammatory response in the context of MDD. The genes *OPRM1*, *EGFR*, and *GSK3B* were selected for their central involvement in the key pathways identified in the functional enrichment analysis (Fig. 1).

## 3 Results

### 3.1 Assembly of MDD gene set and initial enrichment

**Fig. 2:**
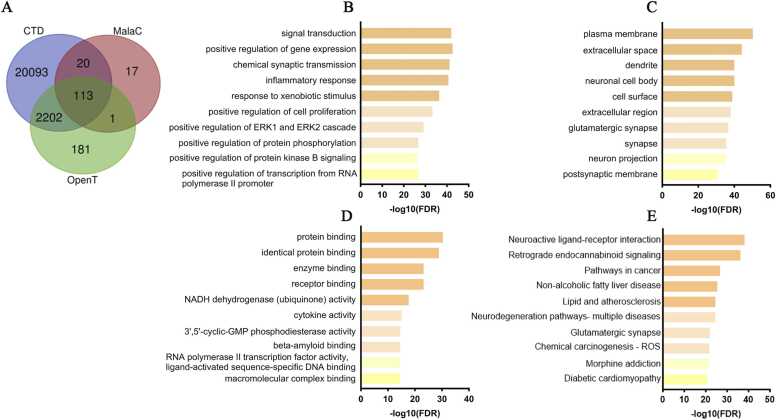
Bioinformatic analysis of genes associated with major depressive disorder (MDD). (A) Venn diagram showing the intersection of genes obtained from CTD, MalaCards, and Open Targets. (B) The top 10 biological processes, sorted by -log10(FDR) using DAVID, including signal transduction, positive regulation of gene expression, and inflammatory response. (C) The 10 most enriched cellular compartments, including plasma membrane, extracellular space, dendrites, and glutamatergic synapse. (D) The 10 most enriched molecular functions by DAVID, including protein binding, NADH dehydrogenase activity, cytokines, and beta-amyloid binding. (E) KEGG pathway analysis highlighting enriched components such as neuroactive ligand-receptor interactions, retrograde endocannabinoid signalling, cancer pathways, and neurodegeneration. Abbrs: SwissP: Swiss Target Prediction; TargetN: TargetNetwork; SEA: Similarity Ensemble Approach.

We compiled genes associated with MDD from CTD, MalaCards, and Open Targets. CTD returned 22,428 genes, MalaCard 151, and Open Targets 2497. After unifying identifiers and removing duplicate, we retained 2336 genes present in at least two sources (Fig. 2A). This intersected set was submitted to functional enrichment with DAVID across Gene Ontology categories and KEGG pathways.

In the biological process category (Fig. 2B), the top enriched terms included “signal transduction”, “positive regulation of gene expression”, “chemical synaptic transmission”, “inflammatory response”, “response to xenobiotic stimulus”, “positive regulation of cell proliferation”, “positive regulation of extracellular signal-regulated kinases 1 and 2 (ERK1 and ERK2) cascade”, “positive regulation of protein phosphorylation”, “positive regulation of protein kinase B signaling”, and “positive regulation of transcription from RNA polymerase II promoter” (Suppl. table 1).

For cellular compartments (Fig. 2C), the most enriched terms were “plasma membrane”, “extracellular space”, “dendrite, neuronal cell body”, “cell surface”, “extracellular region”, “glutamatergic synapse”, “synapse”, “neuron projection”, and “postsynaptic membrane” (Suppl. Table 2).

For molecular functions (Fig. 2D) the leading terms were “protein binding”, “identical protein binding”, “enzyme binding”, “receptor binding”, “nicotinamide adenine dinucleotide (NADH) dehydrogenase (ubiquinone) activity”, “cytokine activity”, “cyclic guanosine 3′,5′-monophosphate (3′,5′-cyclic-GMP) phosphodiesterase activity”, “beta-amyloid binding”, “RNA polymerase II transcription factor activity”, “ligand-activated sequence-specific DNA binding”, and “macromolecular complex binding” (Suppl. Table 3).

KEGG analysis of the same MDD set showed enrichment for “neuroactive ligand-receptor interaction”, “retrograde endocannabinoid signaling”, “pathways in cancer”, “non-alcoholic fatty liver disease”, “lipid and atherosclerosis”, and “pathways of multiple neurodegeneration diseases”, “glutamatergic synapse”, all of which are intrinsically related to the neurobiological mechanisms and pathology of MDD (Fig. 2E; Suppl. Table 4).

### 3.2 Predicted targets for each drug and definition of overlap sets

#### 3.2.1 Fluoxetine druggable targets

**Fig. 3:**
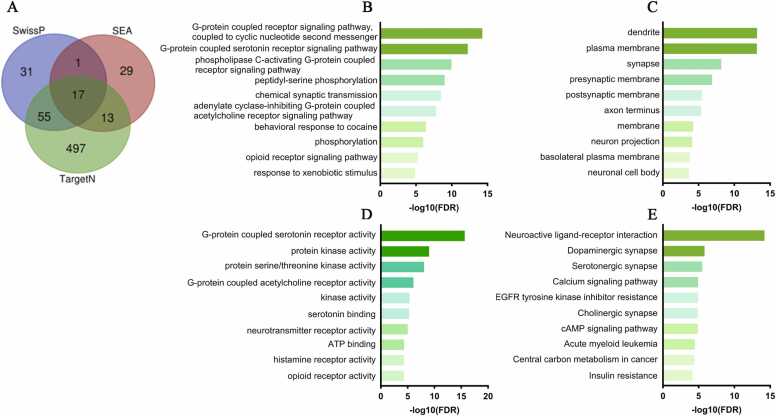
Functional analysis of genes and their potential targets of fluoxetine. (A) Venn diagram showing the 86 shared genes identified in Swissprediction, SEA, and TargetNetwork. (B) Top 10 biological processes enriched in DAVID, highlighting GPCR-mediated signalling and phosphorylation cascades as the most active. (C) Top 10 enriched cellular compartments, with a predominance in dendrites and plasma membrane, as well as synapses, pre- and postsynaptic membranes. (D) Top 10 molecular functions, highlighting ”G-protein coupled serotonin receptor activity” and “G-protein coupled acetylcholine receptor activity”, along with quinase and neurotransmitter binding activities. (E) KEGG analysis of fluoxetine targets, showing enrichment in “Neurosactive ligand-receptor interaction”, dopaminergic synapses, calcium and cAMP signalling pathways, as well as pathways associated with insulin resistance and common mechanisms in oncological contexts. Abbrs: SwissP: Swiss Target Prediction; TargetN: TargetNetwork; SEA: Similarity Ensemble Approach.

At least 86 genes are shared across two or more databases (Fig. 3A), and these were evaluated using DAVID. For the biological process category (Fig. 3B), the most enriched terms reflect a strong emphasis on G protein-coupled receptor (GPCR)-mediated signaling and the regulation of phosphorylation cascades. For example, the "G-protein coupled receptor signaling pathway, coupled to cyclic nucleotide second messenger" pathway featured 13 genes involved, demonstrating robust activation of secondary signaling cascades, essential for the transduction of extracellular signals into intracellular responses (Suppl. Table 5).

A particularly relevant finding is the enrichment of the term “G-protein coupled serotonin receptor signaling pathway,” which involves 9 genes. This reinforces the hypothesis that modulation of the serotonergic system, the main therapeutic target of fluoxetine, is key to its antidepressant effects. Furthermore, terms such as “phospholipase C-activating G-protein coupled receptor signaling pathway” (12 genes), “peptidyl-serine phosphorylation” (13 genes), and “chemical synaptic transmission” (14 genes) (Suppl. Table 5) suggest that fluoxetine may influence several processes related to phosphorylation and synaptic transmission. These findings highlight the complexity of the mechanisms involved in MDD, indicating that fluoxetine not only modulates neuronal activity but also intervenes on multiple fronts to regulate responses to external stimuli and intracellular signaling pathways.

In the cellular compartment analysis (Fig. 3C), our results indicate that fluoxetine targets are predominantly located in key regions for neuronal communication and integration. The term “dendrite,” with 21 enriched genes, suggests that fluoxetine’s effects are concentrated in neuronal extensions where synaptic integration occurs. This finding is reinforced by the enrichment of the term “plasma membrane,” with 57 genes (Suppl. Table 6), highlighting the relevance of cell surface receptors, which are essential for the detection and transduction of extracellular signals. Furthermore, terms such as “synapse,” “presynaptic membrane,” and “postsynaptic membrane” were identified, indicating that fluoxetine modulates structures critical for synaptic transmission.

In the molecular function category (Fig. 3D), the results indicate that the term “G-protein coupled serotonin receptor activity” is the most enriched, involving 11 genes and a fold enrichment of 115.1 (Suppl. Table 7). Furthermore, the terms “protein kinase activity” and “protein serine/threonine kinase activity” suggest that the modulation of phosphorylation cascades plays a key role in the transduction of intracellular signals influenced by the drug. Another important finding is the term “G-protein coupled acetylcholine receptor activity”, with a fold enrichment of 164.5 and a log10 (FDR) of 6 (Suppl. Table 7), suggesting a possible interaction with the cholinergic system. Terms such as "serotonin binding" and "neurotransmitter receptor activity" reinforce the drug's direct involvement in neurotransmitter binding and receptor regulation. Finally, additional functions, such as adenosin triphosphate (ATP) binding and activities related to histamine and opioid receptors, demonstrate that the drug acts on a diverse network of receptor and enzymatic mechanisms.

KEGG analysis of fluoxetine targets reveals that the drug modulates multiple pathways integrating neurochemical transmission, metabolic regulation, and intracellular signaling (Suppl. Table 8). First, the “neuroactive ligand-receptor interaction” pathway is the most enriched, involving 27 genes, demonstrating the broader modulation of neuroactive receptors and ligands essential for synaptic communication. The “dopaminergic synapse” and “serotonergic synapse” pathways reinforce the impact on the dopaminergic and serotonergic systems, both crucial for the antidepressant effects of fluoxetine. Furthermore, the “calcium signaling pathway” and “cyclic adenosine monophosphate (cAMP) signaling pathway” suggest that the drug also influences the regulation of second messengers, which are essential for signal transduction and the control of cellular metabolism. The “cholinergic synapse” pathway again indicates a possible contribution of the cholinergic system to drug action. Interestingly, enriched terms such as “EGFR tyrosine kinase inhibitor resistance,” “Acute myeloid leukemia,” and “central carbon metabolism in cancer” suggest that regulatory mechanisms common to diverse pathological contexts may influence cellular homeostasis in MDD. Finally, the term “insulin resistance” indicates a possible modulation of insulin sensitivity, integrating the neurochemical effects of fluoxetine with metabolic processes (Fig. 3E).

### 3.3 Ketamine druggable targets

**Fig. 4:**
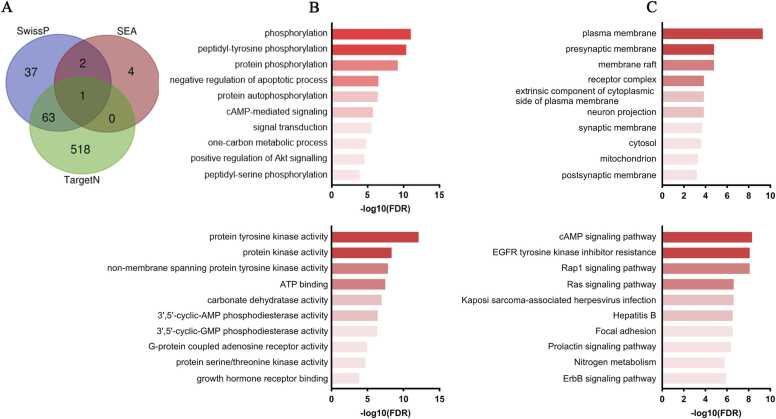
Functional profile of the 66 target genes of ketamine. (A) Venn diagram illustrating the 66 common genes obtained from the databases. (B) Top 10 biological processes, with a predominance of phosphorylation and signalling pathways, as well as second messenger processes and metabolic pathways. (C) Top 10 cellular components, highlighting the plasma membrane, lipid rafts, receptor complexes, as well as terms such as “neuron projection”, “postsynaptic membrane”, and “mitochondria”, evidencing the drug's involvement in signalling structures, neuronal connectivity, and energy metabolism. (D) Top 10 enriched molecular functions, highlighting quinate activity, as well as interactions with growth hormone-related receptors. (E) KEGG analysis reveals the modulation of the ‘cAMP signalling pathway’ as the most significant, along with quinate action, pathways related to infections, cell adhesion and hormonal signalling, and metabolic pathways. Abbrs: SwissP: Swiss Target Prediction; TargetN: TargetNetwork; SEA: Similarity Ensemble Approach.

The Venn diagram shows that 66 genes are shared in at least two databases (Fig. 4A). In the biological process analysis for ketamine targets (Fig. 4B), a strong enrichment in phosphorylation and signaling processes is observed. The term "phosphorylation," with 21 genes, stands out, while "peptidyl-tyrosine phosphorylation" stands out with 10 genes. "Protein phosphorylation" reinforces the role of kinase cascades. "Negative regulation of apoptotic process," with 15 genes, and "protein autophosphorylation" (Suppl. Table 9) suggest protective effects against apoptosis. Additionally, terms such as “cAMP-mediated signaling” and “signal transduction” indicate the modulation of second messenger pathways, while terms such as “one-carbon metabolic process,” “positive regulation of protein kinase B signaling,” and “peptidyl-serine phosphorylation” indicate impacts on metabolic processes and cell survival.

In the analysis of the cellular components of ketamine's druggable targets (Fig. 4C), the results show a strong concentration in membrane structures and neuronal compartments. The term "plasma membrane" is enriched with 45 genes (Suppl. Table 10), indicating the widespread presence of targets in the cellular membrane, where many signaling interactions occur. In contrast, more specialized components such as "presynaptic membrane" and "synaptic membrane" demonstrate high specificity for regions involved in synaptic transmission. Other relevant terms include "membrane raft," which highlights the importance of lipid microdomains in receptor organization, and "receptor complex," which indicates the presence of functional clusters of receptor proteins. Additionally, the term "extrinsic component of the cytoplasmic side of the plasma membrane" reinforces the relevance of the interaction sites on the cytoplasmic side of the membrane. The presence of targets in the neuron projection and postsynaptic membrane highlights the drug's involvement in structures critical for neuronal connectivity and signal transmission. Finally, the contribution of cellular compartments is illustrated by the enrichment in the cytosol and mitochondria, suggesting that metabolic and energy-related processes could also be associated with ketamine's action.

In the molecular function analysis (Fig. 4D), we observed a strong modulation of kinase activities and processes related to intracellular signaling. The term “protein kinase activity,” with 13 genes (Suppl. Table 11), underscores the importance of tyrosine phosphorylation as part of ketamine's mechanism of action. Additionally, “non-membrane spanning protein tyrosine kinase activity” reinforces the role of soluble kinases. Furthermore, “protein kinase activity” confirms the centrality of phosphorylation cascades. “ATP binding” activity highlights the energy dependence of these processes, indicating that ketamine may influence the energy sources required for intracellular signal transduction. Finally, “protein serine/threonine kinase activity” and “growth hormone receptor binding” point to broad enzymatic and receptor regulation, suggesting that these mechanisms could contribute to ketamine's therapeutic effects.

In the KEGG analysis (Suppl. Table 12), the results show the modulation of multiple signaling pathways and metabolic processes. It is important to highlight the term “cAMP signaling pathway” as the most regulated, demonstrating that the regulation of second messengers is crucial for the drug's effects. The term “EGFR tyrosine kinase inhibitor resistance” confirms the previous results, being complemented by the terms “Ras-associated protein 1 (Rap1)” and “rat sarcoma virus protein (Ras) signaling pathway”, which underline the importance of signaling cascades mediated by kinases and small guanosine triphosphatases (GTPases). Furthermore, pathways associated with viral infections, such as “Kaposi sarcoma-associated herpesvirus infection” and “hepatitis B,” may reflect common cellular mechanisms in inflammatory and stress responses (Fig. 4E). Other terms such as "focal adhesion," "prolactin signaling pathway," and "epidermal growth factor receptor (ErbB) signaling pathway" suggest that the interaction between cell adhesion, hormonal signaling, and growth receptors also plays a role in ketamine's action. A potential metabolic effect of the drug is observed in the modulation of pathways such as "nitrogen metabolism," suggesting that ketamine may influence metabolic processes related to cellular nitrogen management.

### 3.4 Esketamine druggable targets

**Fig. 5:**
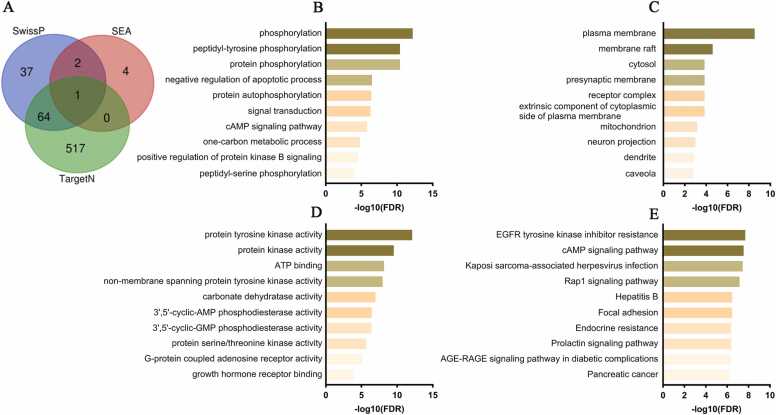
Functional enrichment of the 67 genes and their possible targets of esketamine. (A) Venn diagram illustrating the 67 common genes obtained from the databases. (B) Top 10 biological processes, demonstrating phosphorylation signalling and second messenger pathways. (C) The top 10 cellular compartments again show a predominance of plasma membrane, lipid rafts, pre- and postsynaptic synapses, mitochondria, cytosol, and neuronal projections. (D) Regarding the top molecular functions, highlighting kinases and phosphodiesterase activities as the main molecular mechanisms involved. (E) KEGG analysis shows enrichment in pathways mediated by growth factors (EGFR) and second messengers (cAMP), and pathways related to oxidative damage (AGE-RAGE). Abbrs: SwissP: Swiss Target Prediction; TargetN: TargetNetwork; SEA: Similarity Ensemble Approach.

67 genes are shared in at least two databases (Fig. 5A). Enriched terms for esketamine reveal strong regulation of phosphorylation-mediated signaling (Fig. 5B). Like ketamine, the terms “phosphorylation,” “peptidyl-tyrosine phosphorylation,” and “protein phosphorylation” (Suppl. Table 13) indicate that esketamine strongly modulates the activity of kinases, which are essential for the regulation of synaptic plasticity and neuronal transmission. Furthermore, the presence of the term “negative regulation of apoptotic process” suggests a potent neuroprotective effect, contributing to esketamine's rapid antidepressant effects. The regulation of “cAMP-mediated signaling” and “signal transduction” again highlights the importance of second messenger modulation in drug response. The involvement of the “one-carbon metabolic process” suggests an impact on cellular metabolism, while the “positive regulation of protein kinase B signaling” may indicate an effect on the phosphoinositide 3-kinase (PI3K)/Akt pathway, known for its role in cell survival and neuroplasticity.

Cellular compartments associated with esketamine targets show a predominance of proteins located in the plasma membrane (Fig. 5C and Suppl. Table 14), further reinforcing its role in modulating neurotransmission. The significant presence of terms such as “lipid raft” and “caveola” suggests a role in the organization of lipid microdomains essential for cell signaling. The terms “presynaptic membrane” and “receptor complex” again validate the predicted role of esketamine in synaptic plasticity. The presence of the ketamine-like term “mitochondrion” indicates regulation of neuronal energy metabolism, which may contribute to its rapid effects in the treatment of depression. Interestingly, the terms “cytosol” and “neuron projection” suggest regulation of intracellular proteins associated with synaptic remodeling.

The molecular function of esketamine (Fig. 5D) shows a strong regulation of protein kinase activity, indicating a significant impact on kinase signaling. The high presence of genes involved in ATP binding (25 genes) predicts an interaction of esketamine with energy-dependent processes (Suppl. Table 15). The activity of non-membrane spanning protein tyrosine kinase activity suggests an intracellular modulation of protein phosphorylation. The regulation of carbonate dehydratase activity, unlike ketamine, shows a possible involvement in neuronal acid-base homeostasis. The modulation of phosphodiesterases 3′,5′-cyclic adenosine monophosphate (3′,5′-cyclic-AMP) and 3′,5′-cyclic guanosine monophosphate (3′,5′-cyclic-GMP) suggests an influence of esketamine on cyclic nucleotide-dependent signaling pathways. Furthermore, esketamine appears to interact with G-protein coupled adenosine receptor activity, which may suggest mechanisms of neurotransmission modulation.

The KEGG results are quite similar to those of ketamine (Fig. 5E, Suppl. Table 16). However, in contrast, esketamine showed exclusive involvement in pathways associated with endocrine resistance, glycemic metabolism, and cancer. The term “advanced glycation end-products (AGEs)-receptor for AGEs (RAGE) signaling pathway in diabetic complications” suggests a potential impact of esketamine on oxidative stress and inflammation related to metabolic dysfunction. The term “endocrine resistance” indicates a possible modulation of hormone receptors, which could have implications for the response to hormonal therapies. The association with the term “pancreatic cancer” may suggest interactions with cell proliferation and growth signaling pathways, pointing to pleiotropic effects beyond the CNS.

### 3.5 Shared molecular targets between MDD and drugs

**Fig. 6:**
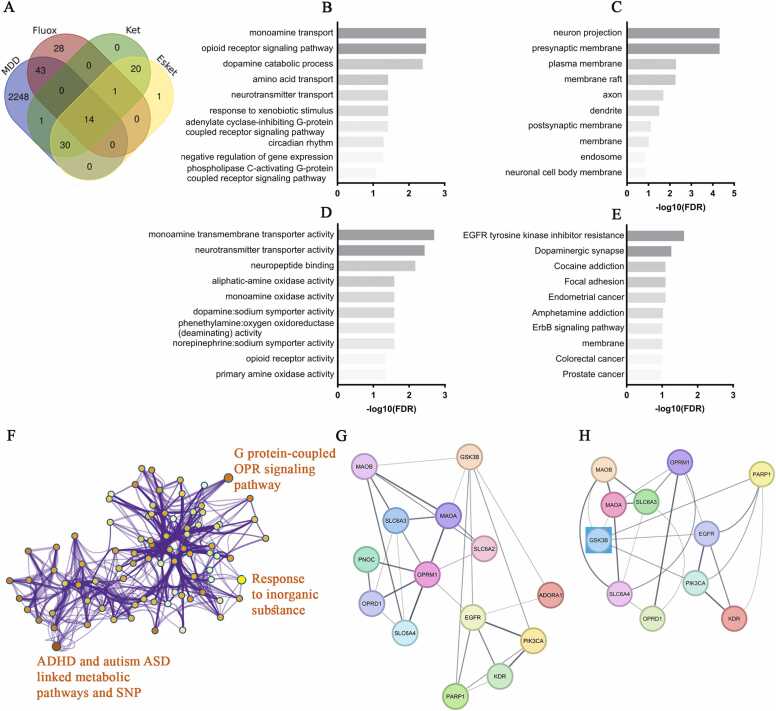
Integrative analysis of the 14 genes shared between MDD and the compounds (fluoxetine, ketamine, and esketamine). (A) Venn diagram showing the 14 genes that appear in common between those associated with MDD and those regulated by the three drugs. (B) Enrichment of shared biological processes, including monoamine transport, catabolic processes, and opioid receptor signalling. (C) Preferential cellular compartments, with emphasis on neuronal projections, presynaptic membranes, axons, dendrites, and lipid rafts. (D) Molecular functions are dominated by monoamine transporter activity, opioid receptor activity, transmembrane transport, neurotransmitter activity, as well as neuropeptide binding and monoamine oxidase activity. (E) Common KEGG pathways, including kinase activity, dopaminergic synapse and signalling, as well as tumour processes. (F) Network of representative terms generated by Metascape, showing the three most significant clusters according to -log (q-valueNote that each term is represented by a circular node, where the size is proportional to the number of genes falling within that term, and the color represents the cluster identity. Terms with a similarity score > 0.3 are unique by an edge, where the thickness of each edge represents the similarity score. When analysing the integrated gene interaction network (G), 14 nodes and 31 edges are observed, connecting MDD genes with drug targets. Using MCODE to identify the most important and critical nodes in the network, *GSK3B* emerges as a potential hub (H). Abbrs: ADHD: attention-deficit/hyperactivity disorder; ASD: autism spectrum disorder, Esket: esketamine: Fluox: fluoxetine; Ket: ketamine; OPR: opioid receptor. Note: Gene names in Table 1.

From the Venn diagram showing the shared genes between those involved in MDD and which, in turn, are regulated or modulated by the drugs, it is observed that 14 genes appear in common (Fig. 6A). The analysis of biological processes between MDD and the drugs (fluoxetine, ketamine, and esketamine) reveals several shared targets involved in common mechanisms (Fig. 6B). One of the main findings is the regulation of the "monoamine transport" target, a crucial process for the transport of neurotransmitters, suggesting a strong association between MDD and the therapeutic effects of these drugs. Another relevant target is the "opioid receptor signaling pathway," which also presents a high fold enrichment value (458.5) (Suppl. Table 17). This is a molecular pathway involved in the regulation of pain, mood, and reward in the brain, which is often altered in MDD.

The term "dopamine catabolic process" was also observed to be highly regulated, suggesting that drugs affect the metabolism of dopamine, a neurotransmitter critical for emotional function and frequently implicated in MDD. Furthermore, processes related to amino acid and neurotransmitter transport, as well as the response to xenobiotic stimuli, are also highly relevant, pointing to a modulation of the cellular environment affected by both MDD and drugs. Finally, the prediction of the term "circadian rhythm" (Suppl. Table 17) suggests a possible effect of drugs on the regulation of biological rhythms, also frequently dysregulated in individuals with MDD, and which could be positively influenced by treatment. The interaction with terms such as "negative regulation of gene expression" and "phospholipase C-activating G-protein coupled receptor signaling pathway" also suggests a convergence in the modulation of gene expression and cell signaling, important elements in the brain's adaptation to the effects of MDD and drug treatment.

For the cellular compartment (Fig. 6C), among the most prominent enriched terms, "neuron projection" suggests a strong relationship with the modulation of neuronal morphology, a fundamental process for communication between neurons that is altered in MDD. Furthermore, "presynaptic membrane" points to a possible action of these drugs on synapses, probably facilitating the release of neurotransmitters. The presence of "axon" and "dendrite" (Suppl. Table 18) reinforces this hypothesis, suggesting the importance of neuronal morphology and synaptic connections in the treatment of MDD, given that these structures are essential for the transmission of electrical and synaptic signals, processes severely affected in the disease. On the other hand, the presence of the terms "plasma membrane" and "membrane raft" again indicates the modulation of signaling and transport processes across the plasma membrane and its specialized domains, which could constitute a key mechanism in the therapeutic effects of these drugs. Other terms such as "endosome" and "neuronal cell body membrane" (Suppl. Table 18) also show significant enrichment, highlighting the importance of modulating intracellular trafficking and cellular homeostasis in the action of these treatments.

The enriched terms in the "molecular function" category (Fig. 6D), based on genes shared between MDD and the analyzed drugs, show a clear focus on the modulation of neurotransmitter transport and monoamine-related enzymatic activities, both fundamental processes in the pathophysiology and treatment of MDD. "Monoamine transmembrane transporter activity" and "neurotransmitter transporter activity" (Suppl. Table 19) evidence a significant regulation of neurotransmitter transport, an essential mechanism for synaptic transmission and the balance of key neurotransmitters such as serotonin, dopamine, and norepinephrine. Furthermore, the presence of terms such as "neuropeptide binding" and "opioid receptor activity" suggests that these drugs may modulate neuropeptide and opioid signaling, pathways that have been implicated in the neurochemical dysfunction of MDD. Our results also highlight the presence of enzymatic activities related to monoamine metabolism, including "monoamine oxidase activity," "dopamine/sodium symporter activity," and "norepinephrine/sodium symporter activity" (Suppl. Table 19), which emerge as key molecular targets of the drugs. These activities are directly involved in neurotransmitter degradation and reuptake, processes that are modulated by antidepressants and that may be crucial for restoring the altered neurochemical balance in MDD.

The KEGG findings reinforce previous observations, showing that EGFR tyrosine kinase signaling pathways, ErbB signaling and dopaminergic synapses, and synaptic vesicle signaling are all within the top 10. Interestingly, there is a predominance of enriched terms related to tumor processes, such as endometrial, colorectal, and prostate cancer, indicating potential roles in tissues outside the CNS (Fig. 6E, Suppl. Table 20). Next, we conducted a meta-analysis using Metascape to identify a subset of terms most representative of the entire cluster and transformed them into an interactive network (Fig. 6F). Our results show that the three most significant (-log(q-value)) clusters are “attention-deficit/hyperactivity disorder (ADHD) and autism spectrum disorder (ASD) linked to metabolic pathways and single nucleotide polymorphisms (SNP) (7.5), response to inorganic substance (4.8), and G protein-coupled opioid receptor signaling pathway (4.6).

### 3.6 Construction and topological analysis of the integrated network

Next, we compiled the molecular targets of the three drugs from databases and the genes associated with MDD. Integrating these data allowed for the construction of an interaction network composed of 14 nodes (including drug targets and genes implicated in MDD) and 31 connections (edges), representing molecular interactions (Fig. 6G). This network encompasses both direct drug targets and genes associated with depression pathology, providing an integrated view of the potential mechanisms involved. To determine the central node of the network and potential functional clusters, we used MCODE, which determined the presence of a cluster with a score of 4800, 11 nodes, and 24 edges, showing *GSK3B* as a potential hub (Fig. 6H).

Topological analysis of the network, based on degree, betweenness, and closeness metrics, reveals its hierarchical organization and the relative importance of each gene in overall connectivity (Table 1). *OPRM1* emerges as the network's main hub, with the highest number of connections (degree = 7), the highest betweenness in the network's paths (betweenness = 0.342), and the highest average proximity to the other genes (closeness = 0.684). These values indicate that *OPRM1* is a central node, facilitating signal transmission within the network and directly influencing its stability and efficiency.

**Table 1:** Topological metrics of genes in the integrated MDD–drug interaction network.

**Gene**	**Name**	**Degree**	**Betweenness Centrality**	**Closeness Centrality**
*OPRM1*	opioid receptor mu 1	7	0.342	0.684
*GSK3B*	glycogen synthase kinase 3 beta	6	0.208	0.590
*EGFR*	epidermal growth factor receptor	6	0.286	0.649
*MAOA*	monoamine oxidase A	6	0.090	0.619
*SLC6A3*	solute carrier family 6 member 3	5	0.022	0.520
*MAOB*	monoamine oxidase B	5	0.070	0.565
*SLC6A4*	solute carrier family 6 member 4	5	0.022	0.520
*PIK3CA*	phosphatidylinositol-4,5-bisphosphate 3-kinase catalytic subunit alpha	4	0.011	0.481
*PARP1*	Poly(ADP-ribose) polymerase 1	4	0.011	0.481
*OPRD1*	opioid receptor delta 1	4	0.017	0.481
*KDR*	kinase insert domain receptor	3	0	0.433
*SLC6A2*	solute carrier family 6 member 2	3	0.005	0.481
*ADORA1*	adenosine A1 receptor	2	0	0.448
*PNOC*	prepronociceptin	2	0	0.433

At a second level of relevance, the genes *GSK3B*, *EGFR*, and monoamine oxidase A (*MAOA)* present a degree = 6 (Table 1). Among them, *EGFR* has a high betweenness value (0.286), suggesting a key role in mediating communication between different parts of the network. *GSK3B*, with a betweenness of 0.208, also acts as a critical point in connectivity, while *MAOA*, despite having the same number of connections, shows a lower impact on mediation (betweenness of 0.090), indicating a lesser influence on information transmission. On the other hand, the genes solute carrier family 6 member 3 (*SLC6A3)* and member 4 (*SLC6A4)* and *MAOB*, with degree = 5, have a lesser influence on network intermediation. Monoamine oxidase B (*MAOB)*, with a betweenness of 0.070, still maintains some control over the flow of information, while *SLC6A3* and *SLC6A4*, with a betweenness of 0.022, have a more peripheral role (Table 1).

At an even lower level, the genes phosphatidylinositol-4,5-bisphosphate 3-kinase catalytic subunit alpha (*PIK3CA)*, poly (ADP-ribose) polymerase 1 (*PARP1)* and opioid receptor delta 1 (*OPRD1)*, with degree = 4, present low betweenness values (< 0.017), suggesting a secondary role in signaling within the network. Finally, the genes kinase insert domain receptor (*KDR)*, solute carrier family 6 member 2 (*SLC6A2)*, adenosine A1 receptor (*ADORA1)* and prepronociceptin (*PNOC)*, with degree ≤ 3, have extremely low or zero betweenness values, indicating that they occupy peripheral positions without significant influence on the overall network structure. From the perspective of closeness, which measures the proximity of a gene to the rest of the network, the most central genes remain *OPRM1*, *EGFR* and *MAOA* (Table 1), reinforcing their fundamental role in connectivity and in the regulation of molecular processes involved in MDD and drug action.

### 3.7 Network fragility analysis

Based on the results of the previous metrics, *OPRM1* was identified as the most critical node in the network, followed by *EGFR* and *GSK3B*, which play key roles in mediating communication. To assess the importance of these genes in network stability, we performed a network fragility analysis, progressively removing each of these genes and examining the impacts on connectivity and overall organization.

**Fig. 7:**
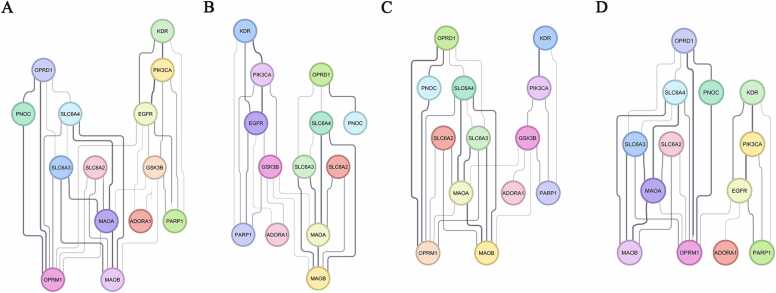
Assessment of network robustness by removing key nodes. (A) Original interaction network comprising 14 genes, displaying a dense and efficient structure with rapid communications between nodes. (B) Removal of *OPRM1* results in pronounced network fragmentation, with loss of connections and increased path lengths between the remaining genes, underscoring its central role in maintaining overall cohesion. (C) Removal of *EGFR* leads to moderate disconnection, characterized by disrupted links and reduced local clustering, although the network retains partial structural integrity. (D) Removal of *GSK3B* causes only mild disruption, with the network largely preserving its connectivity and clustering, though the relative importance of other nodes increases. Note: Gene names in Table 1.

In the original network configuration (Fig. 7A), without gene removal, the network has 14 nodes and 31 links, with a diameter of 3 and an average length of 1.934, indicating efficient communication (Table 2). The clustering coefficient is 0.679, suggesting a high degree of local connectivity, and the heterogeneity is 0.338, indicating a relatively adequate distribution of connections.

**Table 2:** Comparison of topological metrics between the original network and after the removal of key nodes.

**Metric**	**Original network**	**No OPRM1**	**No EGFR**	**No GSK3B**
Number of nodes	14	13	13	13
Number of edges	31	24	25	25
Avg number of neighbors	4.429	3.692	3.846	3.846
Network diameter	3	6	5	4
Network radius	2	3	3	2
Characteristic path length	1.934	2.397	2.244	2.115
Clustering coefficient	0.679	0.636	0.597	0.672
Network density	0.341	0.308	0.321	0.321
Network heterogeneity	0.338	0.374	0.406	0.393
Network centralization	0.231	0.227	0.212	0.311

#### 3.7.1 Impact of *OPRM1* removal

The removal of *OPRM1* (Fig. 7B) results in a reduction in the number of connections (from 31 to 24) and a significant increase in the network diameter (from 3 to 6), demonstrating a significant decrease in communication efficiency (Table 2). The mean path length increases from 1.934 to 2.397, and network heterogeneity rises to 0.374, reflecting a greater imbalance in the distribution of connections. Network density also decreases (from 0.341 to 0.308) (Table 2), indicating a less cohesive structure.

#### 3.7.2 Impact of *EGFR* removal

*EGFR* removal also compromises connectivity (Fig. 7C), but less drastically than *OPRM1*. The network diameter increases to 5, while the average path length grows to 2.244. The clustering coefficient drops to 0.597, indicating a decrease in local interconnectivity (Table 2). Furthermore, heterogeneity increases to 0.406, indicating an increase in the uneven distribution of connections, making the network more vulnerable to the removal of other core nodes.

#### 3.7.3 Impact of *GSK3B* deletion

*GSK3B* deletion (Fig. 7D) causes less disruption to the network compared to *OPRM1* and *EGFR*. Although the network diameter increases to 4, the clustering coefficient (0.672) and mean path length (2.115) remain relatively stable. However, there is a considerable increase in network centralization (from 0.231 to 0.311) (Table 2), indicating that other nodes assume central roles in the network structure after *GSK3B* deletion.

### 3.8 Transcriptional regulation of *OPRM1*, *EGFR*, and *GSK3B*

Analysis of transcription factors in transcriptional regulatory relationships unraveled by sentence-based text mining (TRUUST) reveals that *NFKB* is the only regulator that simultaneously modulates *EGFR*, *OPRM1*, and *GSK3B*, indicating a central role in the interplay between inflammation, synaptic plasticity, and neuronal metabolism in MDD (Table 3). With a highly significant p-value (5.74E-05), *NFKB* regulation of *EGFR* suggests its involvement in epidermal growth factor signaling, which is key to neuroplasticity and neuronal regeneration. Its control over *GSK3B* reinforces its role in kinase modulation, regulating apoptosis and cell proliferation, processes directly linked to neuroinflammation in MDD. Furthermore, *OPRM1* regulation points to a connection between opioid signaling and the inflammatory response, suggesting that modulation of this pathway is critical for the action of antidepressants.

**Table 3:** Transcriptional regulation of key hubs within the network. Abbrev: TF: transcriptional factors.

**TF**	**Name**	**P value**	**Targets**
*NFKB*	nuclear factor kappa B	5.74E-05	*EGFR, ADORA1, GSK3B, OPRM1*
*SP1*	Sp1 transcription factor	0.000317	*EGFR, OPRM1, MAOB, KDR*
*YY1*	YY1 transcription factor	3.80E-05	*EGFR, OPRM1, PARP1*
*SP3*	Sp3 transcription factor	7.25E-05	*MAOB, OPRM1, KDR*
*SP4*	Sp4 transcription factor	2.80E-05	*MAOB, KDR*
*YBX1*	Y box binding protein 1	0.00022	*EGFR, SLC6A4*
*ESR1*	estrogen receptor 1	0.00141	*KDR, EGFR*
*STAT1*	signal transducer and activator of transcription 1, 91 kDa	0.00172	*OPRM1, EGFR*
*STAT3*	signal transducer and activator of transcription 3 (acute-phase response factor)	0.00482	*EGFR, OPRM1*
*JUN*	jun proto-oncogene	0.0053	*EGFR, OPRM1*
*RELA*	v-rel reticuloendotheliosis viral oncogene homolog A (avian)	0.0203	*ADORA1, EGFR*

Other transcription factors that impact *EGFR* and *OPRM1*, such as specificity protein 1 (*SP1)*, Yin Yang 1 (*YY1)*, signal transducer and activator of transcription 1 (*STAT1)* and 3 (*STAT3)* and transcription factor proto-oncogene JUN (*JUN)*, demonstrate the importance of neuronal plasticity and inflammation in the pathophysiology of MDD. SP1 (p = 0.000317) and *YY1* (p = 3.80E-05) regulate genes involved in synaptic remodeling and neuroprotection, while *STAT3* (p = 0.00482) is associated with the inflammatory response and neurogenesis, suggesting its relevance in therapeutic modulation. *JUN* (p = 0.0053), for its part, participates in the response to stress and neuronal apoptosis, reinforcing its link with the synaptic degeneration processes observed in MDD.

## 4 Discussion

Despite therapeutic advances, the molecular basis of treatment-response variability in MDD remains unclear. Our network-pharmacology analysis shows that multiple drugs converge on pathways related to neuroplasticity, cell signaling, and inflammation. We identify GSK3B, OPRM1, and EGFR as central nodes potentially regulated by NFKB, with the integrative identification of OPRM1 and EGFR representing a notable contribution of this work. These findings may help refine existing therapies and support the development of personalized treatments. Moreover, NFKB emerges as a potential candidate biomarker which may serve as an initial basis for exploring more tailored approaches in this disorder.

### 4.1 Analysis of fluoxetine, ketamine and esketamine in MDD: differential modulation of cellular pathways and the central role of NFKB

Integrating multiple databases and retaining genes shared by at least two sources yielded a robust set of 2336 genes, enhancing result reliability and enabling a more precise interpretation of MDD-related biological mechanisms. Functional enrichment analysis highlighted strong involvement of signal transduction, gene-expression regulation, and synaptic neurotransmission, indicating that disrupted cellular communication is a key contributor to MDD pathophysiology. Additionally, enrichment of processes related to cell-proliferation control, the ERK1/2 cascade [52], [53], [54], and Akt signaling [54], [55] suggests modulation of intracellular pathways governing neuronal survival, synaptic plasticity, and stress responses [56], [57]. The activation of inflammatory pathways and xenobiotic-response processes further supports a neuroinflammatory component in MDD [58], e.g., cytokine and β-amyloid-binding activities point to links with neuroinflammation and neurodegeneration, with potential therapeutic implications [32], [59](Suppl. Tables 1–4).

To clarify differences in the activity of each compound, we analyzed their involvement in biological processes, cellular compartments, and key molecular functions, as well as their effects on distinct cellular and physiological mechanisms. This approach provided relevant insights into their therapeutic and secondary effects. Our results support the strong involvement of neuroinflammation in MDD, a finding that coincides with previous studies that report an increase in inflammatory markers, antibodies and lymphocyte infiltration [59]. In line with this, a machine learning study, identified up to 38 relevant genes in MDD some also linked to oxidative stress processes, including *MAPK3* (*ERK1*), whose activation promotes oxidative stress through the production of ROS [60]. Several enriched KEGG pathways were related to cancer. These results likely reflect pleiotropic signaling involved in fundamental cellular processes, such as proliferation and growth, rather than direct mechanistic links to MDD, highlighting shared regulatory mechanisms that may influence cellular homeostasis in MDD, without implying a causal connection to cancer.

Our findings on the transcriptional regulation of key elements in the network through TRUUST analysis showed that *NFKB* (which contains the p50 subunit) is predicted to modulate the transcription of the *EGFR*, *GSK3B* and *OPRM1* genes (Table 3). Previous studies have also shown that *NFKB* can directly bind to and activate the *OPMR1* gene promoter [61], as well as the *EGFR* gene promoter, which contains four KB sites within the promoter [62], although it is not clear whether the regulation between *NFKB* and *EGFR* would be direct [63], [64] or indirect, in *in vitro* studies [65], [66]. Furthermore, our results highlight the role of the S1P as a relevant regulator within the transcriptional network, since it modulates the expression of the genes *EGFR*, *OPRM1*, *MAOB* and *KDR* (Table 3). Previous studies showing Sp1 may acts directly through a subset of NFKB binding sites [67], NFKB and S1P are transcription factors that often interact and collaborate upregulating the expression of crucial genes in the inflammatory immune response [68], [69]. Overall, our results indicate that *NFKB* might have a crucial role as a central regulator of the transcriptional network in MDD, at the level of integration of neuroinflammatory, metabolic and synaptic signals, postulating as a possible candidate biomarker for MDD. In addition, activation of opioid receptors, in particular MOR, has been shown to influence *NFKB* activity, which in turn regulates the expression of various chemokines and pro-inflammatory genes [70].

The potential central role of NFKB as a candidate biomarker for MDD is also supported by several clinical trials that show the role of NFKB in the inflammatory response that occurs in depression [71], [72], [73]. Regarding MOR, numerous clinical and preclinical studies have reported alterations in receptor-mediated signaling in patients with (MDD) as well as in animal models of this pathology [74]. Furthermore, in a recent study conducted in young adult patients with MDD and bipolar disorder (BD), a significant disease-dependent increase in EGF serum levels was observed, suggesting that EGF may represent a potential biomarker for mood disorders [75].

The studied compounds may also regulate neurotransmitters and GABA signalling, e.g., fluoxetine may act directly as an allosteric modulator of GABA-A receptors [76] and 5-HT2A receptor, although this regulation may vary by sex, age, and species [77], [78], [79], [80].

Although the precise mechanisms and the specific neuronal populations mediating its effects remain unclear, ketamine appears to restore homeostatic balance by enhancing synaptic protein synthesis and inducing lasting changes that modulate neuronal microcircuits and promote antidepressant effects [22], [23], [25]. Ketamine and esketamine have demonstrated direct and sustained suppression of the NFKB pathway *in vivo* and *in vitro* models, both in glial and neuronal cells [81], [82], [83], which is associated with a reduction in neuroinflammation and an improvement in depressive symptoms [84]. The differential modulation of studied drugs may partly explain the variability in the onset of action and therapeutic efficacy.

### 4.2 Integrative molecular network analysis identifies OPRM1, EGFR, and GSK3B as key nodes in the therapeutic mechanisms of fluoxetine, ketamine, and esketamine in MDD

The construction of an interaction network integrating molecular targets of fluoxetine, ketamine, and esketamine with MDD-associated genes provided a comprehensive view of the biological mechanisms potentially involved in both pathology and treatment. This network (14 nodes, 31 connections), displayed a non-random architecture, identifying *OPMR1*, *EGFR*, and GSK3B as central nodes and potential regulatory hotspots. MCODE analysis revealed a high-density functional cluster (11 nodes, 4800 score), indicating a subset of highly interconnected genes within the network.

The presence of *GSK3B* as the central node of this cluster strengthen its involvement in mood regulation [85], antidepressant response [86], [87], and inflammatory hypothesis of depression [88]. In addition, topological analysis further refined the functional hierarchy of the genes. *OPRM1* is positioned as the main hub, showing with the highest degree, betweenness and closeness, suggesting a key role in signal transmission and network integration. This finding is particularly relevant given the role of opioid receptors in mood regulation and their emerging involvement in treatments for drug-resistant depression [89]. EGFR, GSK3B, and MAOA formed a second tier of relevance, with EGFR displaying particularly high betweenness, consistent with a bridging node between different functional modules. Although MAOA, despite comparable connectivity, shows lower betweenness, suggesting a more local role. Genes with intermediate connectivity (e.g., SLC6A3, MAOB, SLC6A4) occupy peripheral positions, with MAOB retaining a partial control over information flow, while low-connectivity nodes such as PIK3CA, ADORA1, and PNOC appear marginal, pathological context-dependent and not yet fully characterized.

Network fragility analysis identified OPRM1 as the most critical node, followed by EGFR and GSK3B, as its deletion caused the greatest disruption in network connectivity and organization. Topological analysis confirmed OPRM1 as the main hub, with the highest degree (7), betweenness (0.342), and closeness (0.684), in contrast to OPRD1, which showed lower connectivity (degree = 4) and minimal betweenness (<0.017), indicating a secondary signaling role. Mechanistically, ketamine’s rapid antidepressant and anti-suicidal effects may involve direct opioid receptor signaling in adult MDD patients [90], whereas fluoxetine primarily modulates opioid function indirectly by altering MOR expression and responsiveness rather than through direct receptor binding [91]. These differences suggest convergence at the level of neuronal plasticity but divergence in underlying mechanisms, potentially explaining variability in onset and duration of clinical response, with fluoxetine acting progressively and cumulatively, whereas ketamine/esketamine inducing rapid action in neuronal circuits. Accordingly, modulation of opioid receptors may represent a potential therapeutic strategy for MDD [89], taking sex-related variability into account [92].

Deletion of EGFR and GSK3B also compromised network connectivity. EGFR showed an uneven distribution of connections, whereas GSK3B alteration led to a redistribution of central network roles, resulting in a less pronounced impact than that observed for OPRM1 and EGFR. Taken together, these findings highlight the utility of molecular network analyses for identifying priority nodes in complex biological systems and suggest that genes such as OPRM1, GSK3B, and EGFR may represent strategic targets for more effective and personalized pharmacological treatments in MDD.

## 5 Limitations

This study has several limitations inherent to its computational and meta-analytic design. The analyses rely on publicly available databases, which may be incomplete or biased toward well-characterized genes. Moreover, network and regulatory inferences are predictive and do not establish causality. Finally, experimental validation will be required to confirm the functional relevance of the predicted targets. Accordingly, we propose an experimental approach for NFKB, OPRM1, and EGFR, as central nodes in our network analysis, they can be explored in experimental and clinical studies in MDD: NFKB via peripheral blood mononuclear cells or inflammatory markers (e.g., TNF-α, IL-6) [73], OPRM1 via accessible cells or genetic polymorphisms [74], [93], and EGFR via soluble EGFR or plasma EGF [75]. Differences in the action profiles of fluoxetine, ketamine, and esketamine suggest that each drug targets distinct mechanisms, contributing to variability in patient response. These findings underscore the need for biomarker-guided personalized treatments. Integrating computational and experimental approaches will be essential to advance understanding of MDD mechanisms and optimize novel therapies.

## 6 Conclusions

Our study suggests that the efficacy of drugs in MDD may relate to their ability to modulate key biological processes, including neuroplasticity, cell signaling, and inflammation. The convergence of these drugs on central nodes such as GSK3B, NFKB, OPRM1, and EGFR, together with their specific mechanisms and gradual action, may explain variability in onset and magnitude of clinical response. These nodes represent promising candidate targets for novel or personalized therapeutic strategies, although their predicted roles and regulatory relationships require experimental validation to support biomarker-guided approaches in MDD.

## CRediT authorship contribution statement

**Silvia Tapia González:** Writing – review & editing, Writing – original draft, Validation, Supervision, Resources, Investigation, Funding acquisition, Conceptualization. **George E. Barreto:** Writing – review & editing, Writing – original draft, Validation, Supervision, Resources, Methodology, Investigation, Funding acquisition, Formal analysis, Conceptualization. **Josué García Yagüe:** Writing – review & editing, Writing – original draft, Resources, Investigation.

## Funding

This work was partly supported by funding from Science Foundation Ireland under the Frontiers for the Future Programme (Grant #20/FFPP/8649) to G. E Barreto. This work was also supported by grant FUSP-PPC-24–082, awarded to S. Tapia-González, from Fundación Madri+d, Madrid, Spain, funded by Universidad San Pablo CEU, CEU Universities, Madrid, Spain.

## Declaration of Competing Interest

The authors declare that they have no known competing financial interests or personal relationships that could have appeared to influence the work reported in this paper.

## Data Availability

Data will be made available on request.
