# Supplementary material for: Network pharmacology of cellular targets in major depressive disorder and differential mechanisms of fluoxetine, ketamine and esketamine
Source: Comput Struct Biotechnol J. 2025 Dec 29;31:235–49. doi: 10.1016/j.csbj.2025.12.023 (PMC12810504; doi:10.1016/j.csbj.2025.12.023)
Supplement: Supplementary file 1 — Supplementary material [file mmc1.docx]

**Supplementary material**

**Title: Network pharmacology of cellular targets in major depressive disorder and differential mechanisms of fluoxetine, ketamine, and esketamine**

Silvia Tapia-Gonzalez^a^**^*^**, Josué García Yagüe^a^, George E. Barreto^b^**^*^**

^a^ Grupo de Neurofisiología Celular, Departamento de Ciencias Médicas Básicas, Facultad de Medicina, Instituto de Medicina Molecular Aplicada-Nemesio Díez (IMMA-ND), Universidad San Pablo-CEU, CEU Universities, Urbanización Montepríncipe s/n, Madrid, Spain.

^b^ Department of Biological Sciences, University of Limerick, Limerick, Ireland.

*Corresponding authors.

**Supplementary tables**

| **Term** | **Count** | **Fold enrichment** | **-log10(FDR)** |
| --- | --- | --- | --- |
| signal transduction | 327 | 2.2 | 42.6 |
| positive regulation of gene expression | 182 | 3 | 41.9 |
| chemical synaptic transmission | 114 | 4.2 | 41.1 |
| inflammatory response | 160 | 3.2 | 40.6 |
| response to xenobiotic stimulus | 114 | 3.9 | 36.4 |
| positive regulation of cell proliferation | 167 | 2.8 | 33.2 |
| positive regulation of ERK1 and ERK2 cascade | 98 | 3.7 | 29.1 |
| positive regulation of protein phosphorylation | 92 | 3.7 | 26.6 |
| positive regulation of protein kinase B signaling | 85 | 3.9 | 26.6 |
| positive regulation of transcription from RNA polymerase II promoter | 283 | 1.9 | 26.5 |

Table 1. Top enriched biological processes in MDD.

| **Term** | **Count** | **Fold enrichment** | **-log10(FDR)** |
| --- | --- | --- | --- |
| plasma membrane | 925 | 1.5 | 50.2 |
| extracellular space | 427 | 2 | 44.1 |
| dendrite | 159 | 3.2 | 40 |
| neuronal cell body | 142 | 3.5 | 40 |
| cell surface | 198 | 2.7 | 38.9 |
| extracellular region | 446 | 1.8 | 38 |
| glutamatergic synapse | 148 | 3.2 | 36.6 |
| synapse | 165 | 2.9 | 35.6 |
| neuron projection | 122 | 3.5 | 35.1 |
| postsynaptic membrane | 91 | 4 | 30.9 |

Table 2. Enriched cellular components in MDD-associated genes.

| **Term** | **Count** | **Fold enrichment** | **-log10(FDR)** |
| --- | --- | --- | --- |
| protein binding | 1784 | 1.2 | 30.3 |
| identical protein binding | 376 | 1.8 | 28.8 |
| enzyme binding | 124 | 2.7 | 23.2 |
| receptor binding | 125 | 2.7 | 23.2 |
| NADH dehydrogenase (ubiquinone) activity | 33 | 6.3 | 17.6 |
| cytokine activity | 71 | 3 | 15.1 |
| 3',5'-cyclic-GMP phosphodiesterase activity | 22 | 7.9 | 14.5 |
| beta-amyloid binding | 44 | 4.2 | 14.5 |
| RNA polymerase II transcription factor activity, ligand-activated sequence-specific DNA binding | 34 | 5.2 | 14.4 |
| macromolecular complex binding | 98 | 2.4 | 14.4 |

Table 3. Enriched molecular functions in MDD-enriched genes.

| **Term** | **Count** | **Fold enrichment** | **-log10(FDR)** |
| --- | --- | --- | --- |
| Neuroactive ligand-receptor interaction | 179 | 2.6 | 38.1 |
| Retrograde endocannabinoid signaling | 100 | 3.7 | 36.2 |
| Pathways in cancer | 205 | 2.1 | 26.6 |
| Non-alcoholic fatty liver disease | 90 | 3.1 | 25.3 |
| Lipid and atherosclerosis | 109 | 2.7 | 24.5 |
| Pathways of neurodegeneration - multiple diseases | 185 | 2.1 | 24.5 |
| Glutamatergic synapse | 71 | 3.3 | 22.0 |
| Chemical carcinogenesis - reactive oxygen species | 107 | 2.6 | 21.7 |
| Morphine addiction | 61 | 3.6 | 21.4 |
| Diabetic cardiomyopathy | 99 | 2.6 | 20.7 |

Table 4. Metabolic pathways enriched in MDD according to KEGG analysis.

| **Term** | **Count** | **Fold enrichment** | **-log10(FDR)** |
| --- | --- | --- | --- |
| G-protein coupled receptor signaling pathway, coupled to cyclic nucleotide second messenger | 13 | 56.5 | 14.2 |
| G-protein coupled serotonin receptor signaling pathway | 9 | 132.1 | 12.2 |
| phospholipase C-activating G-protein coupled receptor signaling pathway | 12 | 29.4 | 9.9 |
| peptidyl-serine phosphorylation | 13 | 19.1 | 9 |
| chemical synaptic transmission | 14 | 14.4 | 8.4 |
| adenylate cyclase-inhibiting G-protein coupled acetylcholine receptor signaling pathway | 6 | 176.1 | 7.8 |
| behavioral response to cocaine | 6 | 100.6 | 6.3 |
| phosphorylation | 17 | 6.4 | 6 |
| opioid receptor signaling pathway | 5 | 130.5 | 5.2 |
| response to xenobiotic stimulus | 11 | 10.3 | 4.8 |

Table 5. Top enriched biological processes for fluoxetine-target genes.

| **Term** | **Count** | **Fold enrichment** | **-log10(FDR)** |
| --- | --- | --- | --- |
| dendrite | 21 | 11.9 | 13.1 |
| plasma membrane | 57 | 2.6 | 13.1 |
| synapse | 17 | 8.4 | 8.1 |
| presynaptic membrane | 10 | 18.5 | 6.8 |
| postsynaptic membrane | 10 | 12.5 | 5.4 |
| axon terminus | 7 | 28.7 | 5.3 |
| membrane | 43 | 1.9 | 4.2 |
| neuron projection | 10 | 8.1 | 4.1 |
| basolateral plasma membrane | 9 | 8.6 | 3.7 |
| neuronal cell body | 10 | 6.9 | 3.6 |

Table 6. Cellular components enriched among fluoxetine-target genes.

| **Term** | **Count** | **Fold enrichment** | **-log10(FDR)** |
| --- | --- | --- | --- |
| G-protein coupled serotonin receptor activity | 11 | 115.1 | 15.6 |
| protein kinase activity | 17 | 10.2 | 9 |
| protein serine/threonine kinase activity | 16 | 9.5 | 8 |
| G-protein coupled acetylcholine receptor activity | 5 | 164.5 | 6 |
| kinase activity | 11 | 10.6 | 5.3 |
| serotonin binding | 5 | 104.7 | 5.2 |
| neurotransmitter receptor activity | 6 | 43.2 | 5 |
| ATP binding | 22 | 3.3 | 4.3 |
| histamine receptor activity | 4 | 153.5 | 4.3 |
| opioid receptor activity | 4 | 153.5 | 4.3 |

Table 7. Molecular functions enriched among fluoxetine-target genes.

| **Term** | **Count** | **Fold enrichment** | **-log10(FDR)** |
| --- | --- | --- | --- |
| Neuroactive ligand-receptor interaction | 27 | 8 | 14.2 |
| Dopaminergic synapse | 12 | 9.8 | 5.8 |
| Serotonergic synapse | 11 | 10.4 | 5.5 |
| Calcium signaling pathway | 14 | 6 | 4.9 |
| EGFR tyrosine kinase inhibitor resistance | 9 | 12.3 | 4.9 |
| Cholinergic synapse | 10 | 9.6 | 4.8 |
| cAMP signaling pathway | 13 | 6.3 | 4.8 |
| Acute myeloid leukemia | 8 | 12.9 | 4.4 |
| Central carbon metabolism in cancer | 8 | 12.4 | 4.3 |
| Insulin resistance | 9 | 9 | 4.1 |

Table 8. KEGG pathways enriched among fluoxetine-target genes.

| **Term** | **Count** | **Fold enrichment** | **-log10(FDR)** |
| --- | --- | --- | --- |
| phosphorylation | 21 | 9.8 | 11 |
| peptidyl-tyrosine phosphorylation | 10 | 58.4 | 10.3 |
| protein phosphorylation | 16 | 12.3 | 9.2 |
| negative regulation of apoptotic process | 15 | 8.6 | 6.4 |
| protein autophosphorylation | 10 | 19.6 | 6.4 |
| cAMP-mediated signaling | 7 | 44.4 | 5.7 |
| signal transduction | 20 | 4.7 | 5.5 |
| one-carbon metabolic process | 6 | 50 | 4.7 |
| positive regulation of protein kinase B signaling | 9 | 14.1 | 4.5 |
| peptidyl-serine phosphorylation | 8 | 14.6 | 3.8 |

Table 9. Biological processes enriched among ketamine-target genes.

| **Term** | **Count** | **Fold enrichment** | **-log10(FDR)** |
| --- | --- | --- | --- |
| plasma membrane | 45 | 2.6 | 9.3 |
| presynaptic membrane | 8 | 18.4 | 4.7 |
| membrane raft | 9 | 13.4 | 4.7 |
| receptor complex | 8 | 11.8 | 3.8 |
| extrinsic component of cytoplasmic side of plasma membrane | 5 | 42 | 3.8 |
| neuron projection | 9 | 9.1 | 3.8 |
| synaptic membrane | 5 | 37 | 3.6 |
| cytosol | 35 | 2 | 3.6 |
| mitochondrion | 16 | 3.5 | 3.3 |
| postsynaptic membrane | 7 | 10.8 | 3.2 |

Table 10. Enriched cellular component annotations for ketamine-target genes.

| **Term** | **Count** | **Fold enrichment** | **-log10(FDR)** |
| --- | --- | --- | --- |
| protein tyrosine kinase activity | 13 | 32.9 | 12.1 |
| protein kinase activity | 15 | 11.1 | 8.3 |
| non-membrane spanning protein tyrosine kinase activity | 8 | 49.8 | 7.8 |
| ATP binding | 24 | 4.5 | 7.4 |
| carbonate dehydratase activity | 6 | 101 | 6.9 |
| 3',5'-cyclic-AMP phosphodiesterase activity | 6 | 78 | 6.4 |
| 3',5'-cyclic-GMP phosphodiesterase activity | 6 | 74.6 | 6.3 |
| G-protein coupled adenosine receptor activity | 4 | 228.9 | 4.9 |
| protein serine/threonine kinase activity | 11 | 8.1 | 4.7 |
| growth hormone receptor binding | 4 | 104 | 3.8 |

Table 11. Molecular functions enriched among ketamine-target genes.

| **Term** | **Count** | **Fold enrichment** | **-log10(FDR)** |
| --- | --- | --- | --- |
| cAMP signaling pathway | 16 | 9.5 | 8.3 |
| EGFR tyrosine kinase inhibitor resistance | 11 | 18.6 | 8.1 |
| Rap1 signaling pathway | 15 | 9.5 | 8.1 |
| Ras signaling pathway | 14 | 7.9 | 6.6 |
| Kaposi sarcoma-associated herpesvirus infection | 13 | 8.9 | 6.6 |
| Hepatitis B | 12 | 9.9 | 6.5 |
| Focal adhesion | 13 | 8.5 | 6.5 |
| Prolactin signaling pathway | 9 | 17.1 | 6.3 |
| Nitrogen metabolism | 6 | 47 | 5.9 |
| ErbB signaling pathway | 9 | 14.1 | 5.7 |

Table 12. KEGG pathways enriched for ketamine associated genes.

| **Term** | **Count** | **Fold enrichment** | **-log10(FDR)** |
| --- | --- | --- | --- |
| phosphorylation | 22 | 10.3 | 12.1 |
| peptidyl-tyrosine phosphorylation | 10 | 58.4 | 10.4 |
| protein phosphorylation | 17 | 13.1 | 10.4 |
| negative regulation of apoptotic process | 15 | 8.6 | 6.4 |
| protein autophosphorylation | 10 | 19.6 | 6.3 |
| signal transduction | 21 | 4.9 | 6.2 |
| cAMP-mediated signaling | 7 | 44.4 | 5.7 |
| one-carbon metabolic process | 6 | 50 | 4.7 |
| positive regulation of protein kinase B signaling | 9 | 14.1 | 4.5 |
| peptidyl-serine phosphorylation | 8 | 14.6 | 3.8 |

Table 13. Enriched biological processes for esketamine-target genes.

| **Term** | **Count** | **Fold enrichment** | **-log10(FDR)** |
| --- | --- | --- | --- |
| plasma membrane | 44 | 2.5 | 8.5 |
| membrane raft | 9 | 13.4 | 4.6 |
| cytosol | 36 | 2 | 3.8 |
| presynaptic membrane | 7 | 16.1 | 3.8 |
| receptor complex | 8 | 11.8 | 3.8 |
| extrinsic component of cytoplasmic side of plasma membrane | 5 | 42 | 3.8 |
| mitochondrion | 16 | 3.5 | 3.1 |
| neuron projection | 8 | 8.1 | 3 |
| dendrite | 9 | 6.3 | 2.8 |
| caveola | 5 | 20.7 | 2.8 |

Table 14. Enriched cellular components for esketamine-associated genes.

| **Term** | **Count** | **Fold enrichment** | **-log10(FDR)** |
| --- | --- | --- | --- |
| protein tyrosine kinase activity | 13 | 32.9 | 12.1 |
| protein kinase activity | 16 | 11.9 | 9.5 |
| ATP binding | 25 | 4.6 | 8.1 |
| non-membrane spanning protein tyrosine kinase activity | 8 | 49.8 | 7.9 |
| carbonate dehydratase activity | 6 | 101 | 6.9 |
| 3',5'-cyclic-AMP phosphodiesterase activity | 6 | 78 | 6.4 |
| 3',5'-cyclic-GMP phosphodiesterase activity | 6 | 74.6 | 6.3 |
| protein serine/threonine kinase activity | 12 | 8.9 | 5.6 |
| G-protein coupled adenosine receptor activity | 4 | 228.9 | 5 |
| growth hormone receptor binding | 4 | 104 | 3.8 |

Table 15. Molecular functions represented in esketamine-target genes.

| **Term** | **Count** | **Fold enrichment** | **-log10(FDR)** |
| --- | --- | --- | --- |
| EGFR tyrosine kinase inhibitor resistance | 11 | 18.6 | 7.6 |
| cAMP signaling pathway | 15 | 8.9 | 7.5 |
| Kaposi sarcoma-associated herpesvirus infection | 14 | 9.6 | 7.4 |
| Rap1 signaling pathway | 14 | 8.9 | 7.1 |
| Hepatitis B | 12 | 9.9 | 6.4 |
| Focal adhesion | 13 | 8.5 | 6.4 |
| Endocrine resistance | 10 | 13.6 | 6.3 |
| Prolactin signaling pathway | 9 | 17.1 | 6.3 |
| AGE-RAGE signaling pathway in diabetic complications | 10 | 13.3 | 6.3 |
| Pancreatic cancer | 9 | 15.8 | 6.1 |

Table 16. KEGG pathways enriched among esketamine-target genes.

| **Term** | **Count** | **Fold enrichment** | **-log10(FDR)** |
| --- | --- | --- | --- |
| monoamine transport | 3 | 458.5 | 2.4 |
| opioid receptor signaling pathway | 3 | 458.5 | 2.4 |
| dopamine catabolic process | 3 | 343.9 | 2.3 |
| amino acid transport | 3 | 89.7 | 1.4 |
| neurotransmitter transport | 3 | 89.7 | 1.4 |
| response to xenobiotic stimulus | 4 | 22 | 1.4 |
| adenylate cyclase-inhibiting G-protein coupled receptor signaling pathway | 3 | 75 | 1.4 |
| circadian rhythm | 3 | 60.7 | 1.2 |
| negative regulation of gene expression | 4 | 17.6 | 1.2 |
| phospholipase C-activating G-protein coupled receptor signaling pathway | 3 | 43 | 1 |

Table 17. Biological processes enriched in genes shared between MDD and the drugs fluoxetine, ketamine and esketamine.

| **Term** | **Count** | **Fold enrichment** | **-log10(FDR)** |
| --- | --- | --- | --- |
| neuron projection | 6 | 28.6 | 4.3 |
| presynaptic membrane | 5 | 54.3 | 4.3 |
| plasma membrane | 11 | 3 | 2.2 |
| membrane raft | 4 | 28.1 | 2.2 |
| axon | 4 | 16.6 | 1.6 |
| dendrite | 4 | 13.3 | 1.5 |
| postsynaptic membrane | 3 | 21.9 | 1.1 |
| membrane | 9 | 2.4 | 1 |
| endosome | 3 | 14.2 | 0.8 |
| neuronal cell body membrane | 2 | 101.1 | 0.8 |

Table 18. Cellular components in genes shared between MDD and drugs.

| **Term** | **Count** | **Fold enrichment** | **-log10(FDR)** |
| --- | --- | --- | --- |
| monoamine transmembrane transporter activity | 3 | 404.6 | 2.6 |
| neurotransmitter transporter activity | 3 | 213 | 2.4 |
| neuropeptide binding | 3 | 130.5 | 2.1 |
| aliphatic-amine oxidase activity | 2 | 1348.8 | 1.5 |
| monoamine oxidase activity | 2 | 899.2 | 1.5 |
| dopamine:sodium symporter activity | 2 | 899.2 | 1.5 |
| phenethylamine:oxygen oxidoreductase (deaminating) activity | 2 | 899.2 | 1.5 |
| norepinephrine:sodium symporter activity | 2 | 899.2 | 1.5 |
| opioid receptor activity | 2 | 449.6 | 1.3 |
| primary amine oxidase activity | 2 | 385.4 | 1.3 |

Table 19. Enrichment of molecular functions in genes shared between MDD and drugs.

| **Term** | **Count** | **Fold enrichment** | **-log10(FDR)** |
| --- | --- | --- | --- |
| EGFR tyrosine kinase inhibitor resistance | 4 | 31.3 | 1.61 |
| Dopaminergic synapse | 4 | 18.7 | 1.25 |
| Cocaine addiction | 3 | 37.9 | 1.09 |
| Focal adhesion | 4 | 12.2 | 1.09 |
| Endometrial cancer | 3 | 32 | 1.09 |
| Amphetamine addiction | 3 | 26.9 | 1.02 |
| Synaptic vesicle cycle | 3 | 23.8 | 1.00 |
| ErbB signaling pathway | 3 | 21.8 | 1.00 |
| Colorectal cancer | 3 | 21.6 | 1.00 |
| Prostate cancer | 3 | 19.1 | 0.95 |

Table 20. KEGG pathways enriched among genes shared by MDD and drugs.
